# Definition of the Immune Parameters Related to COVID-19 Severity

**DOI:** 10.3389/fimmu.2022.850846

**Published:** 2022-03-18

**Authors:** Sarah Birindelli, Maciej S. Tarkowski, Marcello Gallucci, Marco Schiuma, Alice Covizzi, Przemysław Lewkowicz, Elena Aloisio, Felicia Stefania Falvella, Alberto Dolci, Agostino Riva, Massimo Galli, Mauro Panteghini

**Affiliations:** ^1^ Clinical Pathology Unit, ASST Fatebenefratelli-Sacco, Milan, Italy; ^2^ Department of Biomedical and Clinical Sciences, “Luigi Sacco”, University of Milan, Milan, Italy; ^3^ Department of Psychology, University of Milano Bicocca, Milan, Italy; ^4^ Department of Infectious Diseases, Division III, ASST Fatebenefratelli-Sacco, Milan, Italy; ^5^ Department of Immunogenetics, Medical University of Lodz, Lodz, Poland

**Keywords:** blood cell count, severity score, immunological changes, COVID-19 outcome, oxygen therapy, clinical management, triage

## Abstract

A relevant portion of patients with disease caused by the severe acute respiratory syndrome coronavirus 2 (COVID-19) experience negative outcome, and several laboratory tests have been proposed to predict disease severity. Among others, dramatic changes in peripheral blood cells have been described. We developed and validated a laboratory score solely based on blood cell parameters to predict survival in hospitalized COVID-19 patients. We retrospectively analyzed 1,619 blood cell count from 226 consecutively hospitalized COVID-19 patients to select parameters for inclusion in a laboratory score predicting severity of disease and survival. The score was derived from lymphocyte- and granulocyte-associated parameters and validated on a separate cohort of 140 consecutive COVID-19 patients. Using ROC curve analysis, a best cutoff for score of 30.6 was derived, which was associated to an overall 82.0% sensitivity (95% CI: 78–84) and 82.5% specificity (95% CI: 80–84) for detecting outcome. The scoring trend effectively separated survivor and non-survivor groups, starting 2 weeks before the end of the hospitalization period. Patients’ score time points were also classified into mild, moderate, severe, and critical according to the symptomatic oxygen therapy administered. Fluctuations of the score should be recorded to highlight a favorable or unfortunate trend of the disease. The predictive score was found to reflect and anticipate the disease gravity, defined by the type of the oxygen support used, giving a proof of its clinical relevance. It offers a fast and reliable tool for supporting clinical decisions and, most important, triage in terms of not only prioritization but also allocation of limited medical resources, especially in the period when therapies are still symptomatic and many are under development. In fact, a prolonged and progressive increase of the score can suggest impaired chances of survival and/or an urgent need for intensive care unit admission.

## Introduction

A new strain of coronavirus is responsible for the outbreak of a pandemic that, by January 6, 2022, has caused 5,462,631 deaths worldwide and reached over 296 million cases of infection (1). The disease caused by severe acute respiratory syndrome coronavirus-2 (SARS-CoV-2), known as coronavirus disease 2019 (COVID-19), is the third documented spillover of an animal coronavirus to humans in the last two decades causing serious disease (2). It created an emergency that, at the beginning, was especially difficult to control in highly prevalent areas, including the Lombardy region in Italy. On February 21, 2020, the first SARS-CoV-2-infected patients were admitted to Luigi Sacco hospital in Milan, one of the two national reference centers for infectious disease. The dramatic increase in new infections and subsequent hospitalizations urgently required a drastic reorganization of the healthcare system, especially the need to admit the growing number of patients in the intensive care unit. The medical emergency continued during patient hospitalization as no effective specific treatment approaches were and are available yet. In the middle of the fourth wave of the COVID-19 pandemic, even though various preventive measures including effective vaccines and improved therapeutic management have been developed, we see the emergence of multiple SARS-CoV-2 variants and a non-reassuring picture of hospital admission rise. Now, as before, SARS-CoV-2-infected patients may be asymptomatic or may present mild symptoms such as fever, dry cough, nausea, asthenia, dysgeusia, anosmia, and myalgia (3–5). About 15% of them, however, can progress to a severe or critical form of the disease with an atypical pneumonia and a progressive respiratory impairment, which can eventually lead to a full-on acute respiratory distress syndrome and an overall fatal outcome (6).

One of the characteristic changes that were observed soon after the pandemic started is the atypical, for the viral infection, distribution of blood cell types in COVID-19 patients especially evident in those who are in severe or critical clinical conditions. Modifications of the number, size, shape, and nuclear and cytoplasmic composition detected in cellular populations of the peripheral blood of COVID-19 patients have been shown to be very dynamic and rapidly occur (7–9). Indeed, among laboratory tests for monitoring hospitalized COVID-19 patients, blood cell count (BCC) is frequently requested and modern hematological analyzers, besides well-known routine parameters, give access to novel ones, potentially useful for rapid monitoring of blood cell changes (10, 11). Brisou et al. (12) in 2014 reported that the LY-Y parameter seems to be crucial in B-cell disorders. Later, in 2017, Fundarena et al. (13) demonstrated the usefulness of lymphocytes’ (LY) positional parameters included in Sysmex XN to differentiate lymphoproliferative disorders. The parameters investigated, LY-X, LY-Y, LY-Z, LY-WX, LY-WY, and LY-WZ, were found to be very useful in detecting disease-associated hematological changes. Recently, several authors documented multi-lineage, morphological changes in circulating blood cells in COVID-19 patients (14). Lymphocytopenia was extensively reported by many as a feature of COVID-19 and a prolonged decline in the absolute LY count has been associated with disease severity and mortality (15). Fahlberg et al. (16) argued if changes in monocyte (MO) or neutrophil (NE) populations precede severe outcomes and if this could direct clinicians to select patients at risk of clinical deterioration. Martens et al. (17) investigated patients with COVID-19 in comparison with COVID-19-negative hospitalized patients affected by respiratory disorders. The former showed both quantitative and qualitative differences in leukocyte populations and a general increase of all hemocytometric markers of activation. In particular, in patients with COVID-19, in addition to an evident imbalance of the LY and NE counts, both cell populations demonstrate enhanced fluorescence signal, which, for NE, is expressed by the NE-SFL (side fluorescent light) parameter, and highly fluorescent lymphocyte cell (HFLC) is the parameter for LY. In both cell populations, enhanced fluorescence activity reflects their status of activation, the measure of which in COVID-19 patients seems to be promising in the prediction of adverse outcome and an independent predictor for mechanical ventilation and death (18). In addition to NE and LY changes, it was observed that in some COVID-19 patients rapid mobilization of neutrophils creates a great demand of new mature cells at the cost of shortening the maturation time of myeloid progenitors (19) and spill-over of immature granulocytes (IG) into the peripheral blood (20).

Overall, this evidence supports a panhemocytometric approach to COVID-19 monitoring: lymphopenia, neutrophilia, and abnormal/activated cells are observed from the onset and appear to have discriminatory capabilities to target patients in mild or critical conditions. More important, their temporal changes may predict disease trajectory (21).

Armed with this knowledge, we have hypothesized if and what peculiar changes of blood cell parameters could be used in the development of the statistical model for monitoring and predicting COVID-19 severity and outcome in hospitalized patients. To confirm or reject the clinical relevance of this scoring model, we sought to determine whether the score can predict, the outcome, as well as the severity of the disease, referred by the type of the oxygen (OXY) therapy (symptomatic treatment) applied.

We show that the statistical model that we developed and the estimated cutoff severity score can be important and valuable elements in the clinical management of COVID-19 hospitalized patients, readily applicable in many diagnostic laboratories equipped with modern hematological analyzers.

## Results

### Characteristics of Study Cohorts

**Table 1** displays demographic and medical history data for the evaluated populations. In addition to the male proportion, significantly lower in the validation cohort, this group showed a longer hospital stay (on average, 3 weeks vs. 2 weeks) and a 2-day later admission to the hospital from symptom onset. For the rest of the characteristics evaluated, no significant differences were found between the two cohorts.

**Table 1:** Baseline characteristics of the evaluated cohorts.

	Development cohort	Validation cohort	*p*-value
***n***	226	140	
**Age** (years) [median (IQR)]	61 (49–72)	61 (50–69)	0.48
**Sex** [*n* (%)]			
Male	154 (68.1%)	66 (47.1%)	<0.001
Female	72 (31.9%)	74 (52.9%)	
**Total *n* of hematological tests**	1,619	1,387	
**Hematological tests per patient** [median (IQR)]	6 (3–9)	6 (4–12)	0.09
**Hospital admission (**days after symptom onset) [median (IQR)]	6 (3–10)	8 (4–11)	0.025
**Hospital stay** (days) [median (IQR)]	15 (9–21)	22 (13–38)	<0.001
**Non-survivors**	49 (21.7%)	40 (28.6%)	0.17
**Comorbidities** [*n* (%)]			
Cardiovascular disease	101 (44.7%)	59 (42.1%)	0.71
Hypertension	76 (33.6%)	51 (36.4%)	0.66
Endocrinopathy	40 (17.7%)	29 (20.7%)	0.56
Diabetes mellitus	30 (13.3%)	26 (18.6%)	0.22
Chronic respiratory disease	25 (11.1%)	14 (10.0%)	0.88
Obesity	10 (4.4%)	12 (8.6%)	0.16
Chronic kidney disease	10 (4.4%)	9 (6.4%)	0.55

IQR, interquartile range.

### Selection of Predictors

**Figure 1:**
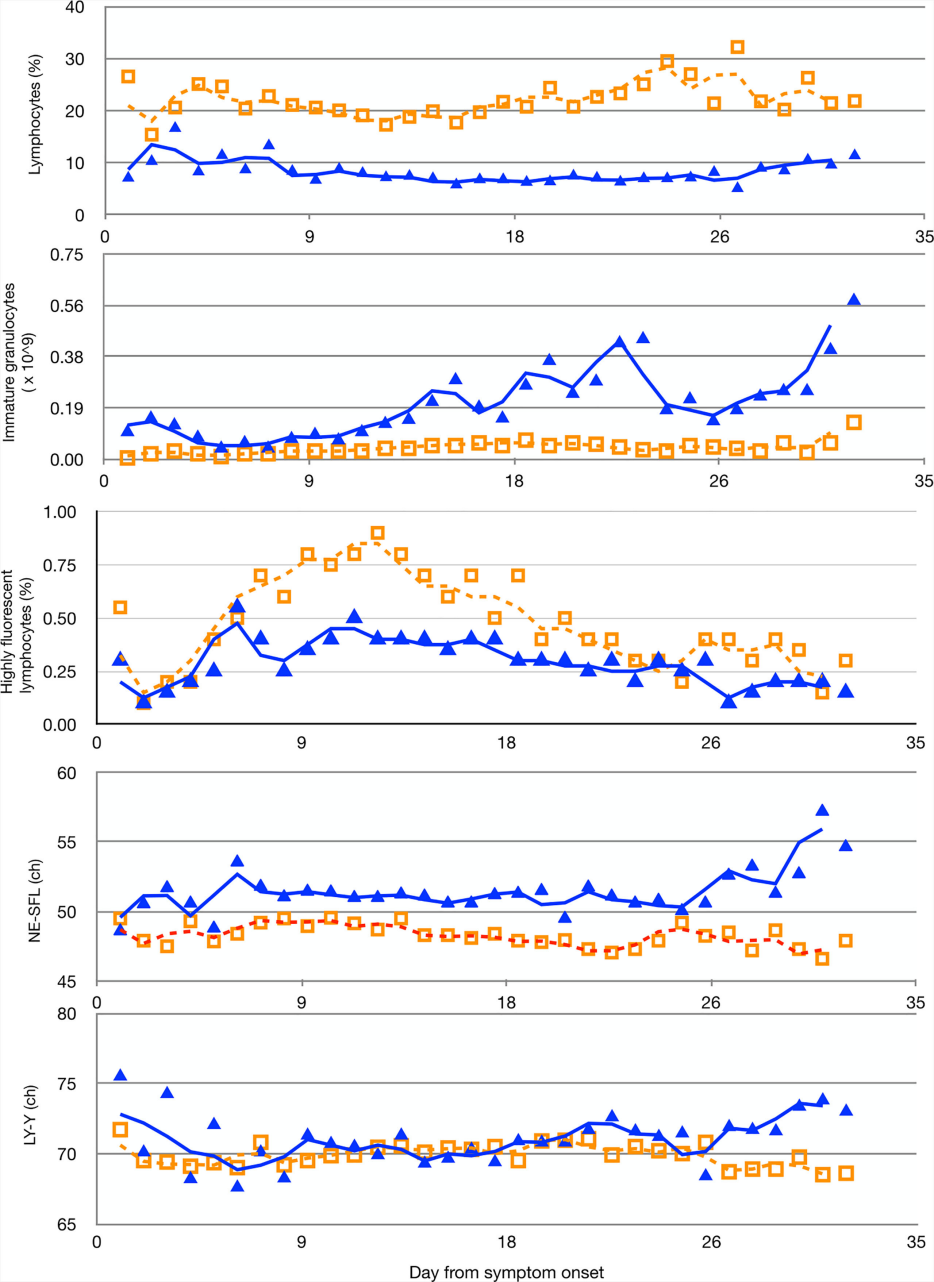
Moving averages of median of multiple measurements of LY%, IG_ABS, HFLC%, the fluorescent light intensity of the neutrophil area on the leukocyte differential (WDF) scattergram (NE-SFL), and the fluorescent light intensity of the lymphocyte area on the WDF scattergram (LY-Y), measured in COVID-19 patients according to days from symptom onset. Solid blue triangles and empty orange squares indicate non-survivors and survivors, respectively. ch, channel-arbitrary units of light scattering.

The final selection included the following parameters: LY%, HFLC%, IG_ABS, NE-SFL, and LY-Y (for a detailed description of the statistical analysis, see **SupplMat 001**). All the selected variables show a peculiar fluctuation over time in patients who survived in comparison with non-survivors (**Figure 1**), and except for HFLC%, all meet statistical significance. LY% is the most significant (*p* < 0.0001) parameter associated with the clinical status of patients during hospitalization. Median values and distribution of WBC-related parameters considered for establishing the model according to COVID-19 outcome are shown in **Table 2**.

**Table 2:** Comparison of all leukocyte-derived parameters evaluated in the study according to the outcome of COVID-19 patients in the development cohort.

Parameters	Survivors (*n* = 177)		Non-survivors (*n* = 49)		p value
	*n* of hematological tests = 1151		*n* of hematological tests = 468		
	Median	IQR	Median	IQR	
WBC (×10^9^/L)	5.73	4.67–7.10	10.71	7.69–14.08	<0.001
NE (×10^9^/L)	3.82	2.58–5.02	9.39	6.36–11.80	<0.001
LY (×10^9^/L)	1.23	1.00–1.63	0.84	0.69–0.99	<0.001
MO (×10^9^/L)	0.50	0.40–0.65	0.46	0.37–0.62	0.21
NE (%)	67.2	57.3–72.9	86.6	81.7–89.3	<0.001
LY (%)	21.7	17.3–30.2	7.4	5.8–11.0	<0.001
MO (%)	8.9	7.3–10.7	4.8	3.4–6.1	<0.001
IG (×10^9^/L)	0.03	0.02–0.06	0.13	0.07–0.34	<0.001
IG (%)	0.6	0.4–0.9	1.5	0.8–2.6	<0.001
HFLC (×10^9^/L)	0.03	0.02–0.05	0.04	0.03–0.05	0.58
HFLC (%)	0.6	0.4–0.9	0.4	0.2–0.6	<0.001
NE-SSC (ch)	151.73	148.50–154.90	151.08	147.31–155.55	0.76
LY-X (ch)	82.00	80.50–83.30	83.15	81.55–84.11	0.001
MO-X (ch)	122.65	121.33–124.14	124.95	123.92–125.98	<0.001
NE-SFL (ch)	48.20	46.52–49.88	50.80	49.45–53.73	<0.001
LY-Y (ch)	69.83	68.30–71.40	70.63	67.80–72.04	0.50
MO-Y (ch)	110.05	107.31–112.77	111.75	108.24–114.40	0.046
NE-FSC (ch)	84.50	82.03–86.29	83.95	81.88–86.60	0.96
LY-Z (ch)	59.78	57.76–60.64	58.95	57.58–60.37	0.31
MO-Z (ch)	62.50	61.40–63.52	62.30	60.00–63.66	0.48

IQR, interquartile range; WBC, white blood cells; NE, neutrophils; LY, lymphocytes; MO, monocytes; IG, immature granulocytes; HFLC, highly fluorescent lymphocyte cells; NE-SSC, the lateral scattered light intensity of the NE area on the WBC differential (WDF) scattergram; ch, channel-arbitrary units of light scattering; LY-X, the lateral scattered light intensity of the LY area on the WDF scattergram; MO-X, the lateral scattered light intensity of the MO area on the WDF scattergram; NE-SFL, the fluorescent light intensity of the NE area on the WDF scattergram; LY-Y, the fluorescent light intensity of the LY area on the WDF scattergram; MO-Y, the fluorescent light intensity of the MO area on the WDF scattergram; NE-FSC, the forward-scattered light intensity of the NE area on the WDF scattergram; LY-Z, the forward-scattered light intensity of the LY area on the WDF scattergram; MO-Z, the forward-scattered light intensity of the MO area on the WDF scattergram.

### Model Derivation, Best Score Cutoff, and Validation of the Predictive Score

Score computing coefficients were based on a logistic regression analysis as follows: linear predictor (LP) = −9.807 + 3.776*IG_ABS − 0.141*LY% − 0.541*HFLC% + 0.224*NE-SFL −0.008*LY-Y, and the score was derived accordingly:


$$score=\frac{1}{1+\exp(-LP)}\cdot 100$$

**Figure 2:**
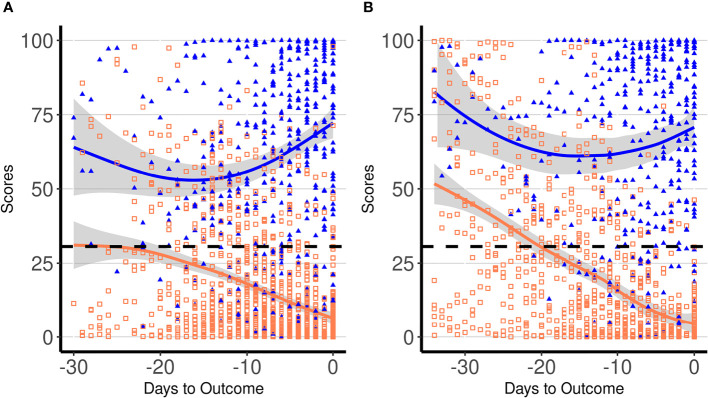
Dynamic profiles of proposed laboratory score in COVID-19 patients according to days to the outcome in the derivation **(A)** and in the validation cohort **(B)**. Symbols indicate single patients’ daily score in survivors (empty orange squares) and non-survivors (solid blue triangles), respectively. Blue and orange lines represent trajectories of daily average score values in non-survivors and survivors, respectively, with the 95% confidence intervals displayed by the shaded area. The dashed line indicates the best cutoff for score (30.6).

The ability of the score to correctly classify patient outcome (survivors vs. non-survivors) was evaluated using all patients’ daily data. At the receiver operating characteristic (ROC) analysis, the area under the curve (AUC) was 0.903 (95% CI: 0.887–0.919), and at a score cutoff of 30.6, a 82.0% sensitivity (95% CI: 78–84) and a 82.5% specificity (95% CI: 80–84) were obtained. The same score cutoff at the day of outcome showed a sensitivity of 95.8% (95% CI: 83–98) and a specificity of 96.0% (95% CI: 92–98). By applying the best cutoff value derived by the ROC curve, we found that the score could start to predict the poor outcome on average 2 weeks before the end of the hospitalization period. **Figure 2A** displays all daily scores for all patients included in the derivation cohort according to the outcome. Since the hospitalization period among patients was different, the daily scores were depicted in relation to the outcome day (death or hospital discharge). The estimated score trajectories for survivors and non-survivors did not overlap, and approximately 2 weeks before the outcome started to diverge. We evaluated the difference between the score trends of the two groups of patients by means of an individual growth model estimated with random-intercept mixed models. Particularly, the significance of the difference between the two curves was estimated as the simple effect of group at −30, −15, and 0 days to outcome. Results confirmed that curves were statistically different (*p* < 0.001) in all three moments [−30, *F*(1, 1,595.3) = 32.9, −15, *F*(1, 365.1) = 37.9, and outcome day, *F*(1, 336.2) = 540.3]. **Figure 2B** displays score results in the validation cohort. In this group, score trajectories did not show statistical difference 30 days before the outcome [*F*(1, 673.5) = 0.11, *p* = 0.742], but showed a marked statistical difference at −15 [*F*(1, 219.5) = 41.6, *p* < 0.001] and at the outcome day [*F*(1, 213.2) = 320.2, *p* < 0.001]. In particular, in the validation cohort, the curves started to become statistically different 24 days before the outcome [*F*(1, 333.7) = 4.1, *p* = 0.044]. Based on these results, the score could effectively predict the patient’s outcome at least 2 weeks before the end of the hospitalization period.

### Analysis of Severity

**Figure 3:**
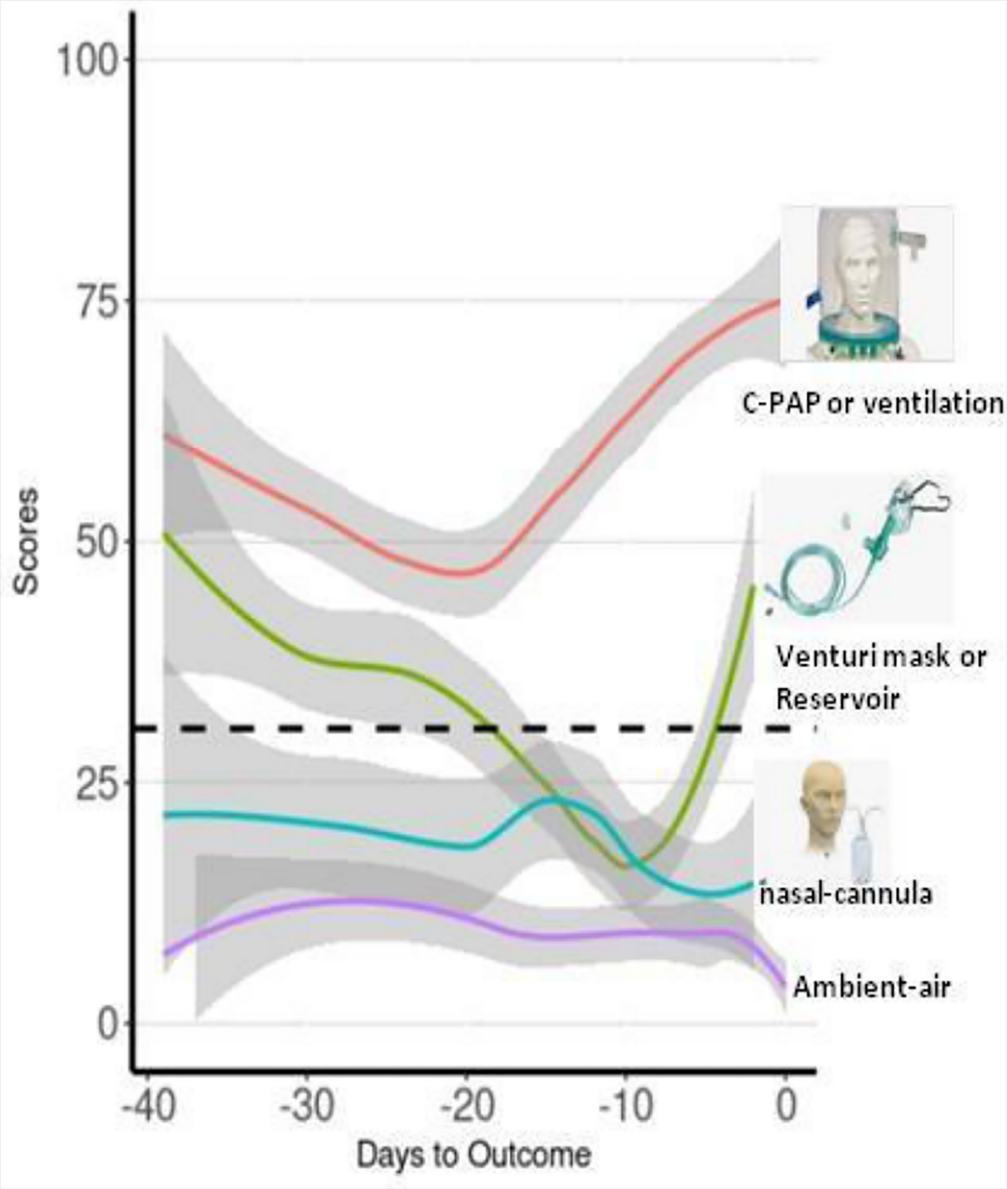
Moving averages of median of score progression over time across severity groups for the assessment of the score values with the severity of patients based on the OXY therapy. Trend lines represent all the time points score measured in patients of the validation cohort according to the OXY therapy. Red line is for “critical” OXY therapy, which is continuous positive airway pressure (CPAP) or mechanical ventilation; green line is for “severe” OXY therapy, which is Venturi mask or reservoir mask; light blue line is for “moderate” OXY therapy, which is nasal-cannula; and purple line is for absence of OXY therapy, which is “mild”.

The score progression over time was compared across severity groups classified into “mild”, “moderate”, “severe”, and “critical” conditions according to the OXY therapy administered by first fitting an individual growth model estimated with random-intercept mixed models (**Figure 3**). The individual growth model was implemented with linear, quadratic, and cubic trend of days to outcome, and their interaction with severity group. The score progression over time was compared across severity groups (for a detailed description of the statistical analysis, see **Supplementary Material**). Groups were compared at 5, 15, and 30 days to outcome, estimating the overall differences due to the group variable. As regards cohort 1, at 5 and 30 days to outcome, we found a severity level overall effect to be statistically significant. As regards cohort 2, at 5 days to outcome, we found a severity level overall effect to be statistically significant and multiple comparisons showed that “critical” was statistically different from all the other three groups (all *p* < 0.001).

## Discussion

In the first months of 2020, the epidemiological scenario of SARS-CoV-2 infection rapidly changed and eventually turned into a pandemic. Soon after the start of the outbreak, it became evident that for proper management of the hospitalized COVID-19 patients, a method was needed to assess the severity of the disease and the outcome. One of the characteristic changes for COVID-19 patients is the atypical, for the viral infection, distribution of cell types in peripheral blood. Taking into the consideration these changes and the availability of conventional and advanced parameters in modern hemoanalyzers, we aimed to develop an easy laboratory score based on both standard and novel hematological parameters to offer a fast predictor of the disease evolution in hospitalized patients and an efficient tool to sort patients into priority groups to determine how best to use scarce resources.

Preliminary data have suggested that the unusually high morbidity and mortality among SARS-CoV-2-positive patients could be associated with the deregulation of host immune responses, the biomarker of which is the dramatic drop of blood LY counts (22). Consistent with the observation that an effective immune response is crucial to counteract the infection, we focused on the hematological parameters reflecting the status of the immune system. To develop the COVID-19 severity prediction model, we took into consideration only the WBC-related parameters known to reflect their activation status or association with infection and/or inflammation: LY%, IG_ABS, HFLC%, NE-SFL, and LY-Y. As shown in **Figure 1**, all the selected variables show a peculiar fluctuation over time in patients who survived in comparison with non-survivors. LY% was found to be the most significant (*p* < 0.0001) parameter associated with the clinical status of patients during hospitalization. It is not a surprise, since LY play a pivotal role in clearing the virus and findings show that SARS-CoV-2-infection can lead to T-cell exhaustion, of which the LY% parameter is the direct reflection (23–25). The detection of IG in the peripheral blood of adults is always associated with the adverse effects of the infection, and it is indicative of an insult to the bone marrow caused by inflammatory reactions (26). We found that from the second week after the onset, IG_ABS was not only always higher in COVID-19 non-survivors in comparison with survivors but peaked several times in the former group. The parameter named HFLC% represents an abnormal cell population placed in the area above the MO and LY region, with high fluorescence intensity. In comparison with normal MO and LY, the increased size and fluorescence, which is a sign of high RNA content, both indicate an “atypical-reactive” population. Their detection in peripheral blood during infectious diseases mirrors the immune response and activation of the immune-competent cells. Previous studies (27, 28) showed the correlation between HFLC and activated B LY, and between HFLC and plasma cells in peripheral blood. By reflecting the activity of B cells, the parameter HFLC% was significantly increased in survivors in the second week after symptom onset, showing the potential for differentiating survivor vs. non-survivor patients.

The evident changes in the HFLC% parameter may reflect not only the intensity of the antibody production but also changes during which B cells become antigen-presenting cells (29). B cells are indeed fundamental in mounting rapid and efficient responses to soluble antigens and in promoting T-cell proliferation and cytokine production. The reason for which there is a lack of statistical significance in HFLC% parameter between survivors and non-survivors is the Cox model we used, which is more sensitive to variations close to the day of outcome. Although, in this way, the relevance of HFLC% parameter was likely to be underestimated in the model, its inclusion is important not only statistically but also biologically. NE are the most abundant circulating WBC and are regarded as the first line of defense of the innate arm of the immune system. However, these cells can also exhibit strong pro-inflammatory reactions if left uncontrolled (30, 31). The formation of granules and vacuoles rich in toxic mediators, closely related to the NE activity, significantly affects cellular changes in complexity and, therefore, the position of the population cluster in the BCC graph distribution, of which the NE-SFL value is the reflection. The signal SFL used by the hemoanalyzer indicates the amount of nucleic acids present in the cell and, as for the parameter HFLC, allows one to distinguish between resting cells from activated cells. Activated cells have a different membrane lipid composition and a greater activity in the cytoplasm, which, in turn, is due to an increase in nucleic acid content that gives a more intense fluorescent staining.

Our data have shown that COVID-19 patients who died experienced a significant increase (*p* < 0.004) in NE-SFL parameter, especially in the last days before death. In addition to the NE-associated parameter, NE-SFL, LY-Y also showed an increase in non-survivors 1 week before the patient’s death. The parameter LY-Y is the equivalent in LY of SFL for NE and, as the last one, it also reflects enhanced nucleic acid synthesis in LY that could be associated with the phenomenon called “cytokine storm” (32). The LY-Y value proportionally increases with the nucleic acid amount, which is the hallmark of activated/abnormal LYs and lymphoblasts, such that Cho et al. proposed this parameter to develop reflex testing rules for screening samples for microscopic examination and to facilitate the detection of abnormal lymphoid cells (33). Thus, it is not surprising that the LY-Y parameter increased the predictive ability of the score when included in the model.

Using the previously described parameters, we developed a model for deriving a laboratory score for predicting COVID-19 severity. By applying the best cutoff value derived by the ROC curve, we found that the score could start to predict the poor outcome on average 2 weeks before the end of the hospitalization period. It should be noted that, in the validation cohort, we found higher score values than the study cohort (**Figure 2**). This can be explained by the time during the pandemic when these patients were admitted to the hospital, i.e., the end of March. By that time, new daily cases in Milan started to suddenly increase, and this trend continued until May 2020. During the same period, thousands of patients were in need of intensive care. In this scenario, in contrast with the previous weeks, more critical cases were admitted to our reference hospital explaining the increase in average score values in hospitalized patients and the longer hospital stay as well as the slight but significant delay in hospital admission. Based on these results, we can argue that our model could effectively predict the patient’s outcome at least 2 weeks before the end of the hospitalization period.

**Figure 4:**
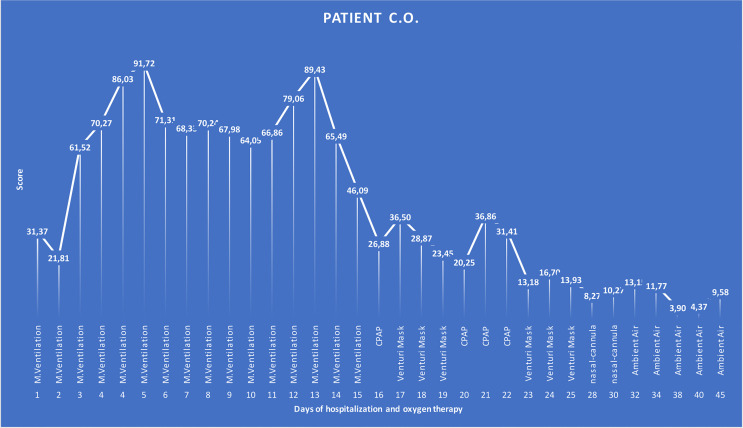
Graphical representation of a COVID-19 patient over the time of 45 days of hospitalization that went through different phases of the disease severity according to the OXY therapy. The patient was considered to be in critical condition upon admission to the hospital and, according to this, supported by CPAP OXY therapy for 16 days (17 score time points—1 day had two BCC and then two scores). After clinical improvement, for the next 9 days (9 score time points), the OXY therapy was alternated between CPAP and a lower grade of support, as Venturi mask is. The patient further improved, and accordingly, the type of the OXY therapy changed to the nasal-cannula on day 28 till 5 days before the discharge when the patient did not require any OXY support. Score values are reported as labels. M. ventilation, mechanical ventilation; CPAP, continuous positive airway pressure.

The aim of our study was to develop a score that can predict not only the final outcome; we sought to develop the score that could be applied as a routine test able to reflect and anticipate the improvement or the worsening of the disease at any moment during the hospitalization. It is known that the most common COVID-19 symptom is dyspnea, which is often accompanied by hypoxemia. Patients with severe disease typically require supplemental OXY and should be monitored closely for worsening respiratory status. Enhanced respiratory support encompasses different OXY strategies from mild to severe according to OXY needs. Most hospitalized COVID-19 patients, in fact, did not have the same level of OXY support needed throughout the whole period of their hospitalization; instead, they went through “critical”, “severe”, “moderate”, and/or “mild” phases according to the symptomatic OXY therapy. In some cases, mild onset evolved into an acute, severe, or critical condition, which eventually improved, leading to a complete recovery, or remained critical until a fatal outcome is reached. As shown in one of the cases retrospectively analyzed (**Figure 4**), over the period of 45 days of hospitalization, the said COVID-19 patient went through different phases of disease severity, each of which had its corresponding type of OXY therapy. The graph shows that for the span of time it took the patient to clinically improve according to the OXY symptomatic therapy, the score was always above the calculated cutoff while it permanently gave values below the cutoff when the OXY symptomatic therapy was reduced. The patient, even if critical for several days, eventually recovered and the score could accurately predict the outcome. We can argue that from day 16, when the score started to be below or near the cutoff, reducing the OXY therapy and avoiding switching between continuous positive airway pressure (CPAP) and Venturi mask after several days could be considered. For the same reason, on day 24, when the score started to be half of the cutoff, further downgrading OXY therapy to the use of nasal-cannula only could be considered.

This conclusion is supported by the results of the analysis of the relation between the OXY therapy and the score we measured in all patients of the validation cohort (**Figure 4**). Interestingly, we obtained four different trend lines that match the type of the OXY symptomatic therapy required, and the score calculated in that phase. It is important to underline that the trend lines do not reflect single specific patients but all the score values of all the patients investigated, classified according to the severity based on the OXY therapy applied. It can be noted that only “mild” and “critical” trend lines reach “day 0”, which represents the day of discharge or death, respectively. “Moderate” and “severe” trend lines stop a few days before the outcome because they reflect a transition to a different phase (lighter or heavier) of the disease, which precedes the outcome. Apparently, the “mild” and “moderate” trend lines show score values constantly below the cutoff, while the “critical” trend line is separate from the other three lines with score values almost always doubling the cutoff. The peculiar shape of the “critical” and “severe” trend lines combines and depicts two different groups of patients. The first half decreasing trend represents those patients who were, from the start, severely ill and who eventually improved and switched to a less aggressive OXY support till total recovery. The second half increasing trend represents those patients who, regardless of the medical approach, remained seriously ill or who suddenly worsened until a fatal outcome is reached. In order to catch these dramatic but very meaningful fluctuations, we did not base the scoring model on only one or a few time point measurements but all the available ones for each patient.

Even if major risk factors for COVID-19 severity have been determined, namely, advanced age, male sex, and presence of comorbidities such as cardiovascular disease, hypertension, diabetes, and obesity (34), when we included age in the model, the prediction ability of the score did not significantly improve. Also, pre-existing pathologies, comorbidities, or drug administration was deliberately not taken into consideration because we showed that the modeled score may provide independent information as it strongly reflects changes in immune-competent cells, which are mainly caused by the virus itself rather than by concomitant clinical conditions. For these reasons, our score has been developed without taking into consideration preexisting pathological conditions or other important variables like age and ongoing therapeutic interventions.

From March 2020, we constantly and repeatedly evaluated the association of the score with clinical conditions of COVID-19 patients admitted to our hospital. As expected, the score was predictive independently of the pandemic waves. In fact, regardless of new emerging variants or the introduction of vaccines, COVID-19 patients continue to suffer from the same respiratory, cardiovascular, renal, digestive, and neuronal virus-related problems, which, in turn, can all be ascribed to uncontrolled immune response (35). Since the combination of cellular parameters on which the score is based can reflect the capability of the immune system to respond to the infections, a high score always reflected a patient in critical condition and a low score always reflected a patient under mild conditions.

**Figure 5:**
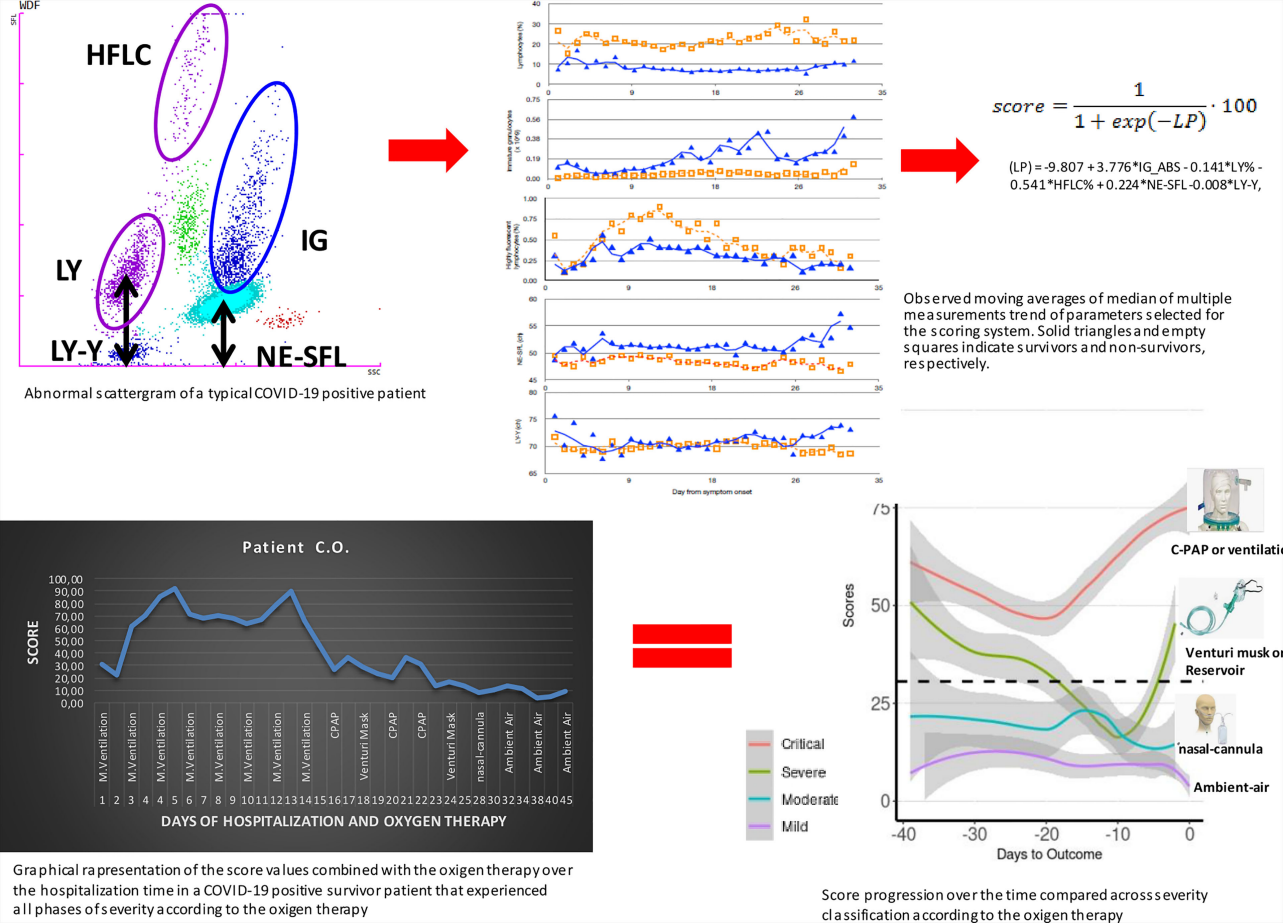
Graphical representation of the entire logical hypothesis of the study starting from the typical abnormal scattergram of a COVID-19-positive patient from which have been selected the 5 predictors of the score. The analysis of data of the five parameters gave the moving averages of median that can clearly show differences between survivors and non-survivors. The accurate statistical analysis provided the final score that has been combined to the severity of patients according to the symptomatic OXY therapy administered. Finally, the combination of all what above described into the graphical representation of a COVID-19 patient over the time of 45 days of hospitalization that went through different phases of the disease severity according to the OXY therapy that show the solid power of the score in representing, preceding, and explaining the course of the disease from the immunological point of view.

Similar to our approach, the use of novel hematological parameters in predicting COVID-19 severity was published (36–44). However, only few authors considered dynamic blood cell changes over different time points crucial in understanding, monitoring, and predicting the severity of the disease. Linssen and co-workers developed a prognostic score based on hematological parameters, but they model the score on the patients’ results during the first 3 days from the admission only to identify critical illness patients irrespective of the final outcome. The aim of our study was to develop a score that, regardless of BCC, could reflect and possibly anticipate any change occurring during the disease and that could modulate and detect the above-described meaningful fluctuations, as well as the final outcome. Additionally, our model combines the chosen variables into an algorithm that eventually releases a score value from 0 to 100, providing clinicians with a modulation of levels of seriousness that can also be translated into different levels of OXY support. In **Figure 5**, we graphically summarize the entire logical hypothesis of the study starting from the typical abnormal scattergram of a COVID-19 positive patient from which have been selected the 5 predictors of the score. The fluctuations of the 5 predictors over the time, have been captured into the score algorithm and the score values have been calculate in all COVID-19 patients investigated. As an example, we show the graphical representation of the score in a COVID-19 patient over the time of 45 days of hospitalization that perfectly matches with the OXY therapy administered. Then we can conclude that the score we derived can precede and reflect the course of the disease from the immunological point of view. Given the above, no scoring systems have been developed to date to monitor daily the disease severity and the chance of survival of hospitalized COVID-19 patients solely based on hematological parameters. The application of the dynamic changes in blood cells that occur during COVID-19 progression and the development of the score that predicts the severity of the disease, as we demonstrated, could help better manage hospitalized patients, and help in the identification of therapeutic interventions and monitoring of their efficacy. In addition to the OXY saturation, the repeated assessment of the score can easily direct clinicians to re-triage patients in order to optimize medical resources. Readily accessible parameters from modern hematological analyzers and the laboratory automation make our test easy to be applied in many laboratories for routine diagnostics. Additionally, it has no additional cost as the extraction of new parameters has already been performed from routinely requested hematological analyses.

## Materials and Methods

### Study Design and Participants

We used BCC data obtained on a SYSMEX XN-series automatic hemoanalyzer acquired from two independent retrospective cohorts to develop and validate a laboratory score model for prediction of the survival and clinical severity in hospitalized COVID-19 patients. For model development, we used data from 1,619 BCCs from 226 COVID-19 patients, consecutively admitted to the L. Sacco hospital from February 21, 2020, to March 29, 2020. SARS-CoV-2 infection was confirmed by reverse transcriptase polymerase chain reaction testing of nasopharyngeal swab. No preexisting pathologies, comorbidities, or therapy administration were considered as exclusion criteria. Data were collected from the electronic hospital database. Medical records were reviewed to confirm the hospitalization outcome and clinical severity. For model validation, a second, independent cohort of 140 consecutive COVID-19 patients, with a total of 1,387 BCCs, was tested. The time point scores of patients from the second cohort were also classified as “mild”, “moderate”, “severe”, and “critical” according to the OXY therapy applied during the hospitalization. More precisely, patients were classified as “mild” when they were normally and autonomously breathing, “moderate” when patients’ oxygenation was supported by nasal-cannula, “severe” when patients’ oxygenation was supported by Venturi mask or reservoir mask, and “critical” when patients’ oxygenation was supported by CPAP or mechanical ventilation. The Institutional Review Board approved the study. To develop the prediction model, we took into consideration only the WBC-related parameters known to reflect cellular changes associated with an infection and/or an inflammation.

### BCC Procedure

All evaluated hematological parameters were measured on peripheral blood samples collected in EDTA-K3 tubes (Beckton Dickinson, Franklin Lakes, NJ, USA), processed within 2 h from the sample collection on a Sysmex XN-series hematology system (Sysmex Co., Kobe, Japan), based on a three-module configuration working in parallel on the same track. Standard and research hematological parameters were collected from the eIPU software for further analyses. The XN platform determines red blood cell and platelet counts, and hematocrit by impedance technology, while white blood cell (WBC) count, leukocyte differential, nucleated red blood cell (NRBC), reticulocyte, and optical platelet counts are measured by flow cytometry. White and nucleated red cell (WNR) channel is used for WBC, NRBC, and basophil counts, whereas the WBC differential (WDF) channel is used for NE, LY, MO, eosinophils, and IG counts. Cells are also classified according to side scattered light (SSC) for cell complexity (NE-SSC, LY-X, and MO-X); side fluorescent light (SFL) for DNA or RNA content (NE-SFL, LY-Y, and MO-Y); and forward scattered light (FSC) for cell size (NE-FSC, LY-Z, and MO-Z). The obtained information based on SSC, SFL, and FSC is related to morphological and functional characteristics of the leukocyte subpopulations, such as cell proliferation and protein production, helpful to monitor blood cells’ response during immuno-inflammatory reactions. In our laboratory organization, the hematology test workflow relies on rule-based technical validation of results by means of a software component provided by Sysmex, named “extended information-processing unit” (eIPU). When fulfilling the rule set validation criteria, results are automatically released to the laboratory information system and then immediately forwarded to the clinical wards. All results not meeting the software-based validation criteria require the supervision of a hematologist who eventually confirms by microscopy the results obtained by the automatic analyzer.

### Model Development

Standard and research hematological parameters were collected from the eIPU software for further analyses. To develop the prediction model, we took into consideration only the WBC-related parameters, known to reflect an infection and/or an inflammatory condition. These parameters included the following: NE absolute count and percentage (NE_ABS and NE%); LY absolute count and percentage (LY_ABS and LY%); MO absolute count and percentage (MO_ABS and MO%); IG absolute count and percentage (IG_ABS and IG%); highly fluorescent LY cell absolute count and percentage (HFLC_ABS and HFLC%); and parameters dealing with morphological and functional characteristics of the WBC subpopulations (NE-SSC, LY-X, MO-X, NE-SFL, LY-Y, MO-Y, NE-FSC, LY-Z, and MO-Z). All the WBC parameters dealing with the dispersion of median values related to the internal complexity (WX), RNA/DNA content (WY), and size (WZ), namely, NE-WX, LY-WX, MO-WX, NE-WY, LY-WY, MO-WY, NE-WZ, LY-WZ, and MO-WZ, were not considered. They denote the dispersion width of the cellular population with regard to size, cellular complexity, and fluorescence intensity, being a marker of coexistence of cells at different stages of differentiation.

### Statistical Analysis

The proposed score was evaluated according to the following outcome: death during hospitalization (non-survivors) vs. hospital discharge after clinical recovery (survivors). Demographic, clinical, and laboratory characteristics were compared between patients classified into these two categories. Data were reported as percentages for categorical variables and median with interquartile range limits for quantitative variables. Differences between variables in different categories were assessed by applying chi-square test (categorical) and Mann–Whitney rank-sum test (quantitative). A Cox proportional hazard model with time-varying covariates was used to investigate the predictive ability of the selected parameters. To reduce the influence of random fluctuations in the parameters, the entire hospitalization period of each patient was divided into three intervals of equal length. Time periods were identified by days of stay for each patient. For patients with length of hospitalization shorter than 1 week, only one interval was defined. The score coefficients were obtained by using a logistic regression with the clinical outcome as dependent variable and the set of markers as independent variables. Logistic regression was used because it yields coefficients that are like the Cox hazard model but offers an easier way to compute a risk score on a daily basis. The overall statistical significance of the model was investigated by the likelihood ratio (LR) test and the Akaike’s information criterion (AIC), the former providing a test of the null hypothesis for the full model, and the latter giving information about the goodness of the fit of the model itself. To understand the stability of the scores, we performed a bootstrap re-sampling approach and computed the bootstrap percentile confidence intervals (CI). Each interval was at 95% confidence, using the 2.5th and 97.5th percentile of the bootstrap distribution obtained with 1,000 bootstrap samples. The best cutoff value for the score for predicting death was obtained from a ROC analysis, by choosing the value that maximized diagnostic accuracy. Trend lines, depicting dynamic changes of the scores calculated per day and per patient in the two groups (survivors vs. non-survivors) of both cohorts, were derived. Differences between the score curves of the two groups of patients were evaluated by an individual growth model estimated with random-intercept mixed models. The individual growth model was implemented with linear and quadratic trend of days to outcome, and their interaction with patient group. The score progression over time was compared across severity groups by first fitting an individual growth model estimated with random-intercept mixed models. The individual growth model was implemented with linear, quadratic, and cubic trend of days to outcome, and their interaction with severity group. Groups were compared at 5, 15, and 30 days to outcome estimating the overall differences due to the group variable. Each overall difference effect at different days to outcome was probed with Bonferroni correction pairwise comparisons. A *p*-value <0.05 denoted statistical significance. All statistical analyses were done using R software, version 3.6.3 (R Foundation for Statistical Computing, Vienna, Austria).

## Data Availability Statement

The raw data supporting the conclusions of this article will be made available by the authors, without undue reservation.

## Author Contributions

SB and MP, conceptualization design of the study. MT, creation of the model. SB, data curation. MGallucci, statistical analysis. MS, AC and AR, patient data. SB, MP, MT, and MGallucci, preparation of tables and figures. SB, MT, PL, EA, FF, AD, and MGalli, data analysis and interpretation. SB and MT, writing original draft. MP, critical revision of the manuscript. All authors critically reviewed the draft and approved the final version for publication.

## Funding

This study was partially supported by University of Milan.

## Conflict of Interest

The authors declare that the research was conducted in the absence of any commercial or financial relationships that could be construed as a potential conflict of interest.

## Publisher’s Note

All claims expressed in this article are solely those of the authors and do not necessarily represent those of their affiliated organizations, or those of the publisher, the editors and the reviewers. Any product that may be evaluated in this article, or claim that may be made by its manufacturer, is not guaranteed or endorsed by the publisher.

## Abbreviations

SARS-CoV-2, severe acute respiratory syndrome coronavirus-2; COVID-19, SARS-CoV-2 disease; BCC, blood cell count; WBC, white blood cells; NRBC, nucleated red blood cells; WNR, white and nucleated red cell; WDF, WBC differential; NE, neutrophils; LY, lymphocytes; MO, monocytes; IG, immature granulocytes; SSC, side scattering light; SFL, side fluorescent light; FSC, forward scattered light; eIPU, extended information-processing unit; NE_ABS, NE absolute count; NE%, NE percentage; LY_ABS, LY absolute count; LY%, LY percentage; MO_ABS, MO absolute count; MO%, MO percentage; IG_ABS, IG absolute count; IG%, IG percentage; HFLC_ABS, highly fluorescent lymphocyte cell absolute count; HFLC%, highly fluorescent lymphocyte cell percentage; OXY, oxygen; CPAP, continuous positive airway pressure CI, confidence interval; ROC, receiver operating characteristic; AUC, area under the curve.
