# Corrigendum: Definition of the immune parameters related to COVID-19 severity

**DOI:** 10.3389/fimmu.2023.1219179

**Published:** 2023-05-24

**Authors:** Sarah Birindelli, Maciej S. Tarkowski, Marcello Gallucci, Marco Schiuma, Alice Covizzi, Przemysław Lewkowicz, Elena Aloisio, Felicia Stefania Falvella, Alberto Dolci, Agostino Riva, Massimo Galli, Mauro Panteghini

**Affiliations:** ^1^ Clinical Pathology Unit, ASST Fatebenefratelli-Sacco, Milan, Italy; ^2^ Department of Biomedical and Clinical Sciences, “Luigi Sacco”, University of Milan, Milan, Italy; ^3^ Department of Psychology, University of Milano Bicocca, Milan, Italy; ^4^ Department of Infectious Diseases, Division III, ASST Fatebenefratelli-Sacco, Milan, Italy; ^5^ Department of Immunogenetics, Medical University of Lodz, Lodz, Poland

**Keywords:** blood cell count, severity score, immunological changes, COVID-19 outcome, oxygen therapy, clinical management, triage

In the published article, Clinical Chemistry and Laboratory Medicine - A panhaemocytometric approach to COVID-19: a retrospective study on the importance of monocyte and neutrophil population data on Sysmex XN-series analysers (10.1515/cclm-2021-0096) was not cited in the article. The citation has now been inserted in the **Introduction** and should read:

“Overall, this evidence supports a panhemocytometric approach to COVID-19 monitoring: lymphopenia, neutrophilia, and abnormal/activated cells are observed from the onset and appear to have discriminatory capabilities to target patients in mild or critical conditions. More important, their temporal changes may predict disease trajectory (21).”

The References section has been updated and renumbered accordingly.

The authors apologize for this error and state that this does not change the scientific conclusions of the article in any way. The original article has been updated.
