# Supplementary material for: Definition of the Immune Parameters Related to COVID-19 Severity
Source: Front Immunol. 2022 Mar 18;13:850846. doi: 10.3389/fimmu.2022.850846 (PMC8971756; doi:10.3389/fimmu.2022.850846)

**Supplementary Material**

*Statistical analysis*

The proposed score was evaluated according to the following outcome: death during hospitalization (non-survivors) vs. hospital discharge after clinical recovery (survivors). Demographic, clinical and laboratory characteristics were compared between patients separated in these two categories. Data were reported as percentages for categorical variables and median with interquartile range limits for quantitative variables. Differences between variables in different categories were assessed by applying chi-squared test (categorical) and Mann-Whitney rank-sum test (quantitative). A Cox proportional hazard model with time-varying covariates was used to investigate the predictive ability of the selected parameters. To reduce the influence of random fluctuations in the parameters, the whole hospitalization period of each patient was divided in three intervals of equal length. Time periods were identified by days of stay for each patient. For patients with length of hospitalization shorter than one week, only one interval was defined. The score coefficients were obtained by using a logistic regression with the clinical outcome as dependent variable and the set of markers as independent variables. Logistic regression was used because it yields coefficients that are like the Cox hazard model but offers an easier way to compute a risk score on the daily basis. The overall statistical significance of the model was investigated by the likelihood ratio (LR) test and the Akaike's information criterion (AIC), the former providing a test of the null hypothesis for the full model, and the latter giving information about the goodness of the fit of the model itself. To understand the stability of the scores, we performed a bootstrap re-sampling approach and computed the bootstrap percentile confidence intervals (CI). Each interval was at 95% confidence, using the 2.5^th^ and 97.5^th^ percentile of the bootstrap distribution obtained with 1000 bootstrap samples. The best cut-toff value for the score for predicting death was obtained from a receiver operating characteristic (ROC) analysis, by choosing the value that maximized diagnostic accuracy. Trend lines, depicting dynamic changes of the scores calculated per day and per patient in the two groups (survivors vs. non-survivors) of both cohorts, were derived. Differences between the score curves of the two groups of patients were evaluated by an individual growth model estimated with random-intercepts mixed models. The individual growth model was implemented with linear and quadratic trend of days to outcome, and their interaction with patient group. The score progression over time was compared across severity groups by first fitting an individual growth model estimated with random-intercepts mixed models. The individual growth model was implemented with linear, quadratic and cubic trend of days to outcome, and their interaction with severity group. Groups were compared at 5, 15 and 30 days to outcome estimating the overall differences due to the group variable. Each overall difference effect at different days to outcome was probed with Bonferoni correction pairwise comparisons. A p value <0.05 denoted statistical significance. All statistical analyses were done using R software, version 3.6.3 (R Foundation for Statistical Computing, Vienna, Austria).

*Selection of predictors*

All the WBC-related parameters not included into the score, NE_ABS, NE%, MO_ABS, MO%, and LY_ABS, showing a large redundancy with LY%, indicated by the elevated correlation with this parameter (Spearman correlation coefficients ≥0.75), were not considered for further testing. Also, the morphological and functional characteristics of MO, namely MO-X, MO-Y, and MO-Z, were not further considered.

The model built with LY% (z=-7.138, p <0.0001), LY-Y (z=-2.166, p=0.03), NE-SFL (z=2.894, p=0.004), IG_ABS (z=-2.006, p=0.045), and HFLC% (z=1.507, p=0.13) was tested with a Cox proportional hazard model, resulting good enough (LR=128.8, p<0.0001, AIC=659.7).

A set of Cox hazard models were estimated for the remaining WBC parameters not included in the model (NE-SSC, LY-X, NE-FSC, LY-Z), adding each parameter one at a time. However, the addition of none of them improved the model fit in a statistically significant way.

*Analysis of severity*

The score progression over time was compared across severity groups by first fitting an individual growth model estimated with random-intercepts mixed models. The individual growth model was implemented with linear, quadratic and cubic trend of days to outcome, and their interaction with severity group. Groups were compared at 5, 15 and 30 days to outcome estimating the overall differences due to the group variable. Each overall difference effect at different days to outcome was probed with Bonferoni correction pairwise comparisons.

As regards cohort 1, at 5 days to outcome we found a severity level overall effect to be statistically significant,

F(3,1506.384)=34.138,p.<.001. Multiple comparisons showed that critical was statistically different from all the other three groups (all p.<.001). , Severe was different from Mild (p.=0), whereas Moderate was not different from Severe (p.=0.183) and from Mild (p.=0.154). At 15 days to outcome we found a severity level overall effect to be statistically significant, F(3,1119.735)=19.383, p.<.001. Multiple comparisons showed that critical was statistically different from all the other three groups (all p.<.001), whereas the other three groups were not statistically different (all p.>.14). At day 30 to outcome, we found a severity level overall effect to be statistically significant, F(3,1386.523)=5.741,p.<.001. The only differences were due to Critical vs Moderate, p.=.0002, and Severe vs Moderate, p.=.005. The other groups were not different (all p.>.08).

Cohort 2

Severe was different from Mild (p.=0.009), whereas Moderate was not different from Severe (p.=0.407) and from Mild (p.=1). At 15 days to outcome we found a severity level overall effect to be statistically significant, F(3,1069.022)=17.599,p.<.001. Multiple comparisons showed that critical was statistically different from all the other three groups (all p.<.001), and Severe was different from all other three groups (p.<.016). Moderate vs Mild did not differ significantly (p.=1.00). At day 30 to outcome, we found a severity level overall effect to be statistically significant, F(3,1014.973)=5.574,p.<.001. The only differences were due to Critical vs Moderate, p.=.024, and Critical vs Severe, p.=.005. The other groups were not different (all p.>.32).

Additionally, the score progression over time was compared across severity groups by selecting patients’s scores at a specific day to end, and by conducting a one-way ANOVA with pairwise comparisons t-tests with Bonferroni correction. Groups were compared at 5, 15 and 30 days. Due the small sample of scores at 30 days to outcome, for those comparisons scores with days to outcome more than 25 days were considered.

**Cohort 1**


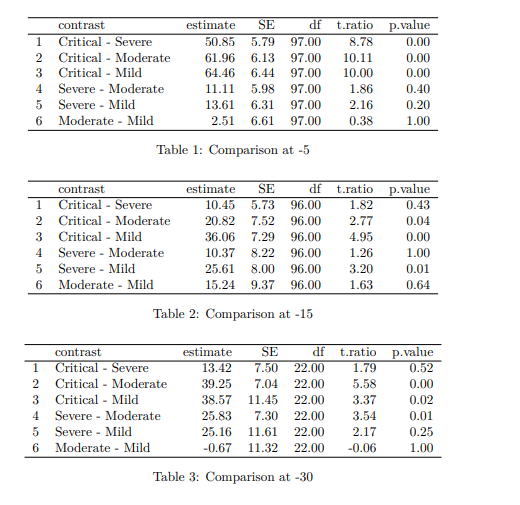


**Cohort 2**


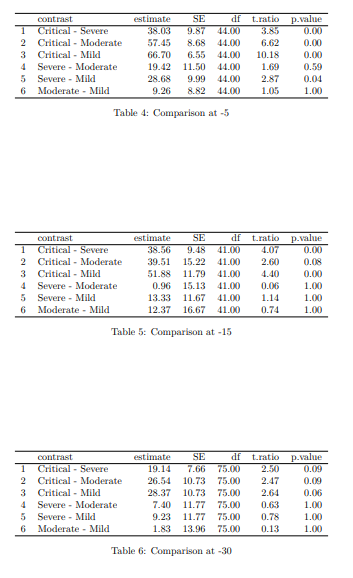

Supplement: Supplementary file 1 [file DataSheet_1.docx]
